# Deep learning-based bacterial genus identification

**DOI:** 10.5455/javar.2022.i626

**Published:** 2022-11-18

**Authors:** Md. Shafiur Rahman Khan, Ishrat Khan, Md. Abdus Sattar Bag, Machbah Uddin, Md. Rakib Hassan, Jayedul Hassan

**Affiliations:** 1 Department of Computer Science and Mathematics, Faculty of Agricultural Engineering & Technology, Bangladesh Agricultural University, Mymensingh, Bangladesh; 2 Department of Computer Science and Engineering, Faculty of Agricultural Engineering and Technology, Khulna Agricultural University, Khulna, Bangladesh; 3 Department of Microbiology and Hygiene, Faculty of Veterinary Science, Bangladesh Agricultural University, Mymensingh, Bangladesh

**Keywords:** Convolutional neural network, deep learning, digital image bacterial genera, automated bacteria identification

## Abstract

**Objectives:**

This study aimed to develop a computerized deep learning (DL) technique to identify bacterial genera more precisely in minimum time than the usual, traditional, and commonly used techniques like cultural, staining, and morphological characteristics.

**Materials and Methods:**

A convolutional neural network as a part of machine learning (ML) for bacterial genera identification methods was developed using python programming language and the Keras API with TensorFlow ML or DL framework to discriminate bacterial genera, e.g., *Streptococcus*, *Staphylococcus*, *Escherichia*, *Salmonella*, and *Corynebacterium.* A total of 200 digital microscopic cell images comprising 40 of each of the genera mentioned above were used in this study.

**Results:**

The developed technique could identify and distinguish microscopic images of *Streptococcus*, *Staphylococcus*, *Escherichia*, *Salmonella*, and *Corynebacterium* with the highest accuracy of 92.20% for *Staphylococcus* and the lowest of 77.40% for *Salmonella*. Among the five epochs, the accuracy rate of bacterial genera identification of *Staphylococcus* was graded 1, and *Streptococcus*,* Escherichia*,* Corynebacterium*, and* Salmonella* as 2, 3, 4, and 5, respectively.

**Conclusion:**

The experimental results suggest using the DL method to predict bacterial genera included in this study. However, further improvement with more bacterial genera, especially of similar morphology, is necessary to make the technique widely used for bacterial genera identification.

## Introduction

Microorganisms are crucial in various areas of life, although some of them (infectious ones) are the causes of various diseases. Thus, identifying microorganisms is crucial in medical science to suggest targeted or specific treatment regimens. Microbiologists, especially bacteriologists, have traditionally identified bacteria using cultural, morphological, staining characteristics, molecular techniques like polymerase chain reaction (PCR), and molecular phylogenetic methods (e.g., sequencing of the *16S rRNA* gene). However, some techniques, like PCR and sequencing, are more costly and time-consuming and are not necessary in many cases. Instead, tracking the morphology or visual characteristics of the bacteria is the priority. From this perspective, the automation of the bacterial identification system would help reshape the process and prompt decision-making in disease prevention, diagnosis, and treatment [1]. A subset of machine learning techniques called deep learning (DL) is ideally suited for teaching algorithms to distinguish between different bacteria based on appearance [1]. For bacterial identification, artificial neural networks were utilized successfully [2]. Using geometric features and a variation of the Naive Bayes method, the identification of bacterial cells using digital microscopic cell images has just been introduced [3]. Technology has begun to be used more widely.

Bacterial genera identification using light microscopy and other bacterial genera identification techniques can be time-consuming and requires well-trained bacteriologists. Gram stain, cultural, and biochemical examinations are routinely performed for bacterial genus identification [4]. Although some of these tests are very prompt, complete identification takes hours to days depending on the organisms, especially fastidious ones. This hampers patient management by selecting suitable strategies, especially antibiotics and supportive care. Therefore, a rapid and accurate technique for identifying routinely encountered bacteria is warranted [5].

The prerequisites for disease diagnosis and control plans are proper and early detection, reporting, and early prevention and control of any specific diseases caused by bacterial genera. The bacteriologists identify and diagnose the diseases using traditional instruments and techniques for the staining and morphological studies made by microscopic studies. As a part of these diagnostic techniques and a plan of research techniques, computer-based analytic tools can be used for the more successful and more effective bacterial genus identification in the diagnostic purpose of any specific disease (personnel communication). For example, computer digital image processing, Machine Learning (ML) models, and other convolutional neural networks (CNN) DL, a branch of ML, can be used as a bio-computing technique for various imaging techniques in the proposed study. Supervised ML techniques, particularly CNN, are becoming more popular across various industries and offer unmatched accuracy in picture classification tasks [6]. The application of CNN with massive labeled datasets has been made possible over the past 10 years by advancements in computing technology, especially Graphical Processing Unit technology, scientific research, and user-friendly software like TensorFlow [7], Theano [8], Keras [1], and Torch [9]. CNN outperforms conventional image processing techniques thanks to its quick picture classification capabilities, and they need a lot of manually labeled data to train the network. With only minor drawbacks, ML techniques have been effectively applied to biological optical microscopy data [10,11]. DL has been used to identify bacterial genera from 2D images [12] and divide images down to the pixel level [13].

In this article, we propose a strategy for the Digital Image Bacterial Genera (DIBaG) technology that uses high-level characteristics that capture morphological motifs for the automatic identification and categorization of bacterial genera. In contrast to earlier studies using other microorganisms, this work relied on DL to identify high-level traits that distinguish the various bacterial genera in morphological aspects instead of hand-designing the features. In the first stage, namely, a supervised DL technique called CNN was used to separate bacterial genera colonies and staining traits based on morphological parameters. The suggested DL framework was tested using actual photos from the bacteriology lab of the Department of Microbiology and Hygiene. This study described the first application of the DL framework for learning high-level characteristics of bacterial genera identification both domestically and internationally, which will act as a starting point for analyzing changes in colony morphology caused by interactions between various bacterial genera.

## Materials and Methods

### Bacterial strains and images

Microscopic images of five morphologically distinct bacterial genera, including *Corynebacterium*, *Escherichia*, *Salmonella*, *Staphylococcus,* and *Streptococcus,* were used in this study. Among them, bacteria strains belonging to *Escherichia*, *Salmonella*, *Staphylococcus,* and *Streptococcus* were revived from the repository of the Department of Microbiology and Hygiene, Bangladesh Agricultural University (BAU), Mymensingh (Table 1). The bacterial strains were revived from the glycerol stock onto Luria Bertani agar (Liofilchem, Italy). Single isolated colonies from each of the bacterial strains were subjected to Gram’s staining [14] and examined using a microscope (Olympus CX31 microscope equipped with SC30 camera, Olympus^(R)^, Japan) under 100× objectives with oil immersion. A total of 160 images, including 40 images from each of the four genera described above, were taken with a resolution of various pixels. Images of the genus *Corynebacterium* were collected from websites and publications described earlier [15–17].

**Table 1.** Bacterial strains used in this study with their source and staining characteristics.

Bacterial strains	Source (No.), host	Gram’s staining characteristics	References
*Escherichia*	Mastitic milk (19), cattle	Gram-negative, non-spore-forming rods arranged singly or in pairs or short chain	[18]
	Rectal swab (21), cattle		[19]
*Salmonella*	Feces (20), cattle	Gram-negative, non-spore-forming rods arranged singly or in pairs	[20]
	Feces (20), ducks		[21]
*Staphylococcus*	Mastitic milk (40), cattle	Gram-positive cocci, arranged in clusters	[19] (Unpublished)
*Streptococcus*	Mastitic milk (40), cattle	Gram-positive cocci, arranged in paired and/or chains	[19] (Unpublished)
*Corynebacterium*	-	Gram-positive, slender rod, club-shaped in appearance	[15–17]

### Feeding and processing of data

**Figure 1.**
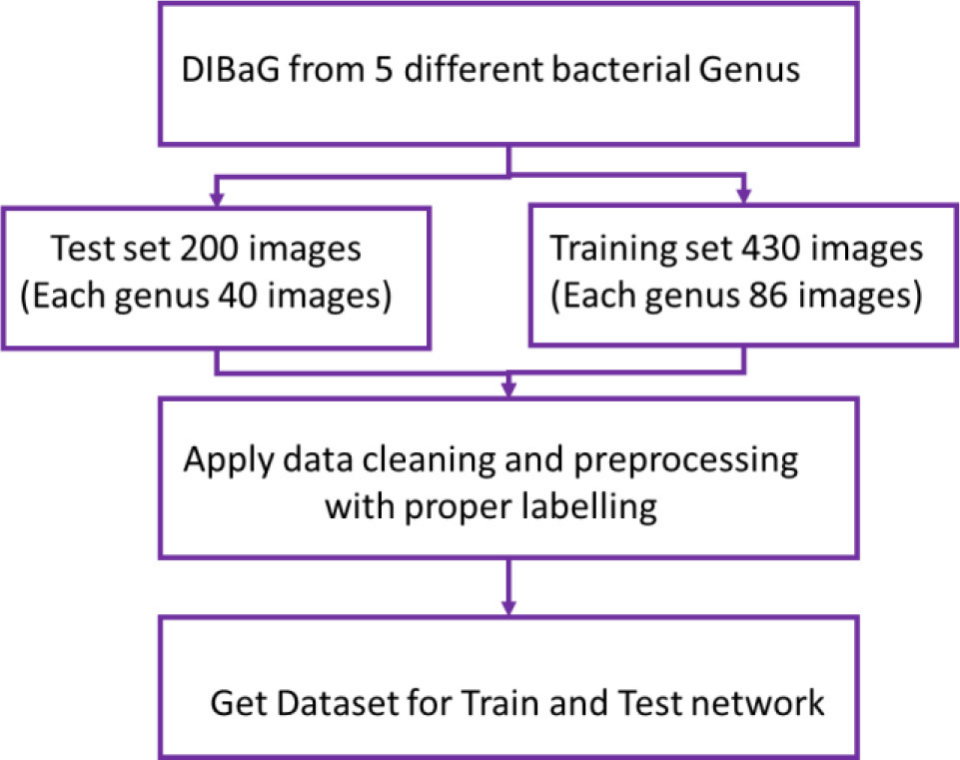
Feeding and processing of data set as testing and training set in to the deep learning network.

**Figure 2.**
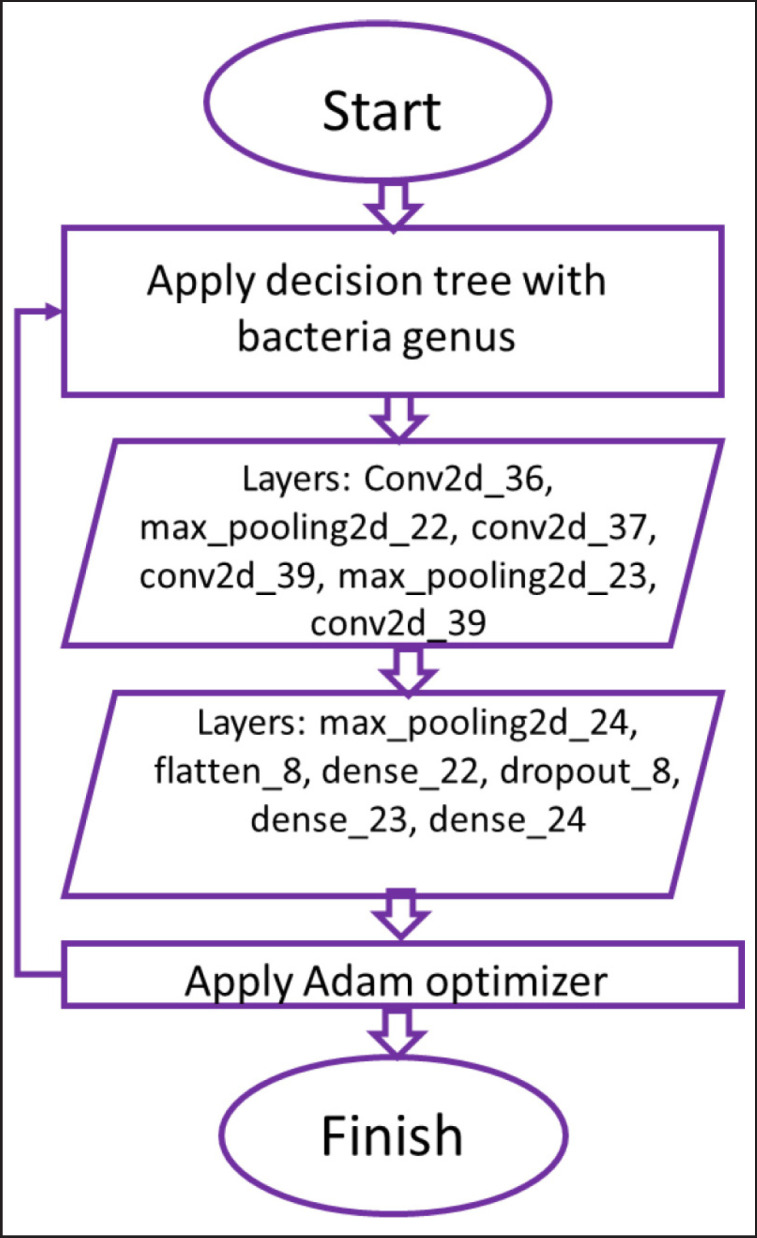
Schematic illustration of the experimental design describing bacterial genera identification by DL as flowchart of the designed methodology of computer-aided technology.

The bacterial genera images were resized to fixed pixels as per the requirements where the essential features such as edges and clusters were considered. Images were DIBaG, followed by the preparation of test and training datasets (Fig. 1). After proper labeling, all selected datasets were fed into the CNN, where the convolution layer was comprised of a 3 × 3 filter. The following 13 layers of conv2d, max pooling2d, flatten 8, dense 22, dropout 8, and Adam optimizer, the DL technique was trained (Fig. 2) using a decision tree derived from the DIBaG *Corynebacterium, Escherichia, Salmonella, Staphylococcus* and S*treptococcus*. Here, the input was a bacteria image learned using a 2D convolution layer. The convolution layer converted input image data to concise information and learned the network using the mathematical operations of median filters. Then it applied the max pooling layer. Thus, it reduced the size of the input image. Later, a softmax function was used to flatten layer data. In the next step, the method uses a dropout layer for removing overfitting problems as well as optimizing the learning. The process continues 13 times, using 13 different layers. To achieve faster results, the method employs the Adam optimizer. Moreover, this function can work well with fewer parameters tuned into CNN networks. Thus, the method identified the bacterial genus type.

### Dataset

In this study, five different sample sets were used. These sample sets are taxonomically and morphologically distinct bacterial genera of three Gram-positive bacteria (*Corynebacterium, Staphylococcus and Streptococcus*) and two genera of Gram-negative bacteria (*Escherichia* and *Salmonella*) (Table 1). We prepared a test set of 200 digital images (each genus 40 images) and a training set of 430 images (each genus 86 images).

## Results and Discussion

In this DL-based DIBaG investigation, the bacterial genera *Corynebacterium, Escherichia, Salmonella, Staphylococcus and Streptococcus*, were investigated utilizing high-level features that capture morphological motifs in digital images. After consideration, data (200 images) were collected from the bacteriology laboratory of the Department of Microbiology and Hygiene, BAU, Mymensingh, and web-based resources (for *Corynebacterium*) for the whole study. In contrast to earlier studies with other microorganisms, this study utilized DL to find high-level features that characterize the various bacterial genera stated above based on their morphological properties rather than hand-designing the features. In particular, CNN was used in the initial phase to differentiate between the bacterial species based on morphological characteristics. Then real-data experiments were conducted to test the suggested DL framework. The scientific contributions of this work were: 1) the first application of the DL framework to learn high-level features of bacterial genera of* Corynebacterium, Escherichia, Salmonella, Staphylococcus* and* Streptococcus* identification in the context of home and abroad; and 2) the high-level features are being provided as a basis for the analysis of colony morphology changes and staining reactions induced by the cross-bacterial genera interactions.

### Feeding analysis as training size and data augmentation

**Figure 3.**
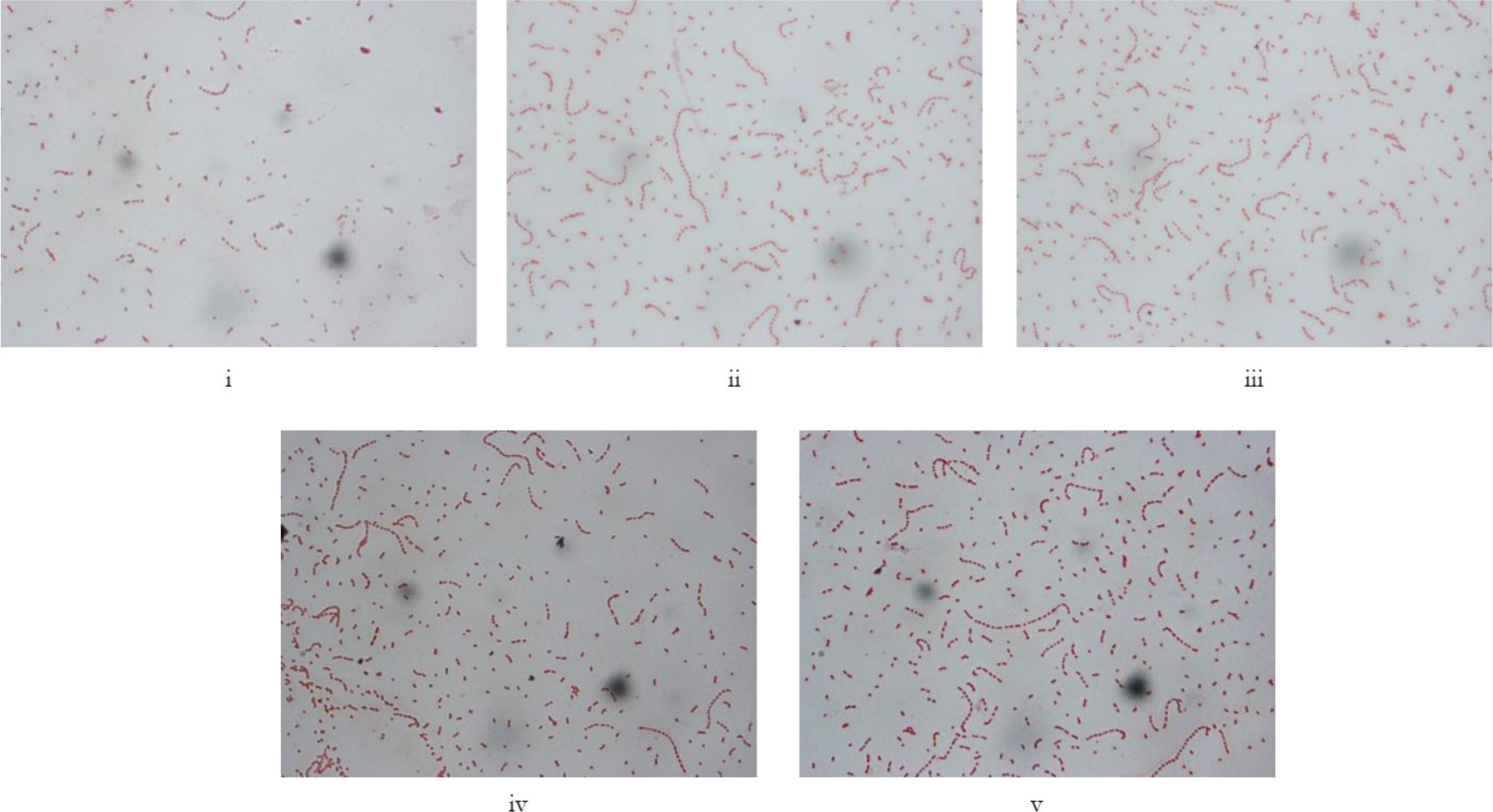
(i–v) Representative images of Gram staining and morphological characteristics of the genus *Streptococcus* (100×). *Streptococcus* are gram-positive bacteria arranged in short or long chains, non-spore-forming, and non-flagellated.

**Figure 4.**
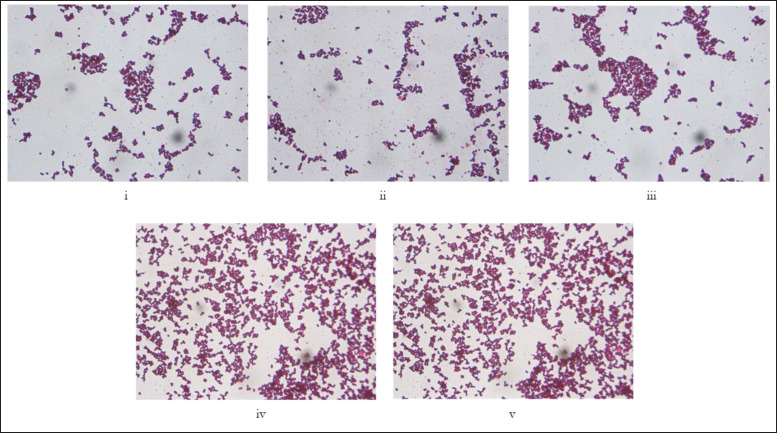
(i–v) Representative images of Gram staining and morphological characteristics of the genus *Staphylococcus* (100×). The genus *Staphylococcus* is gram-positive and morphologically spherical and arranged in grape-like clusters. They do not produce spores or carry any flagella.

**Figure 5.**
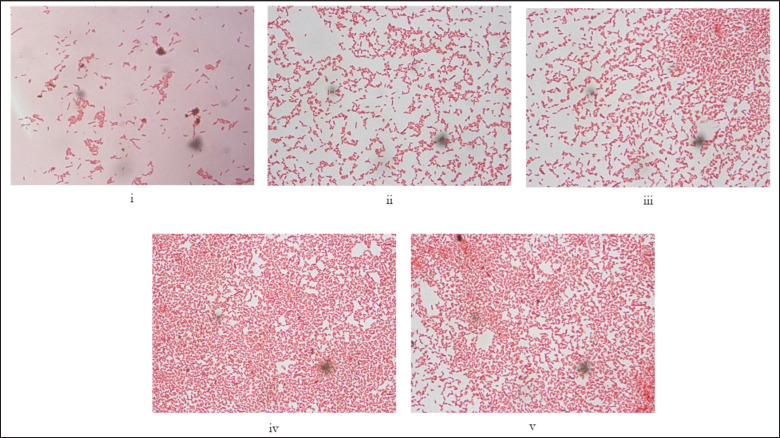
(i–v). Representative images of Gram staining and morphological characteristics of the genus *Escherichia* (100×). *Escherichia* are gram-negative rods arranged singly or in pairs. They carry peritrichous flagella but do not produces spore.

**Figure 6.**
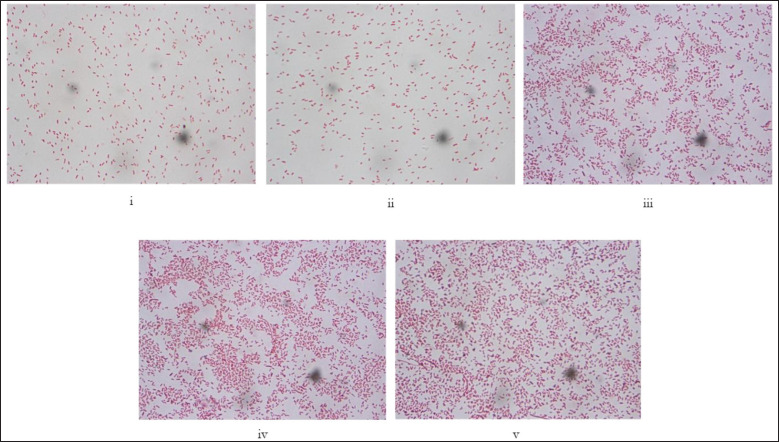
(i–v). Representative images of Gram staining and morphological characteristics of the genus *Salmonella* (100×). *Salmonella* is gram-negative, shaped like a short bar, arranges singly or in pairs, and mostly carries peritrichous flagella but no spore.

**Figure 7.**
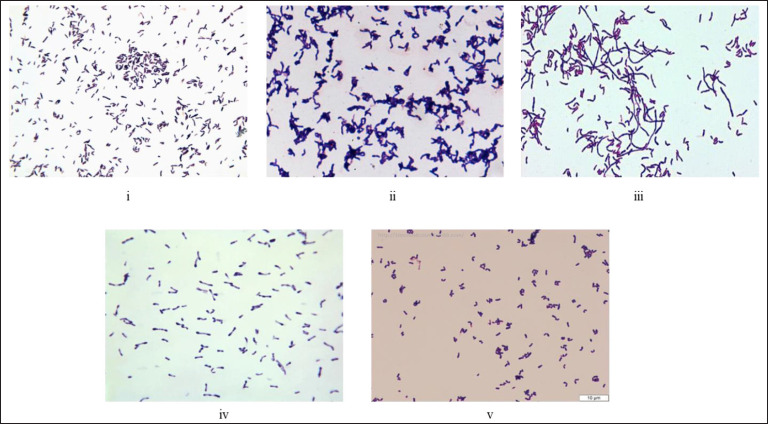
(i–v). Representative images of staining and morphological characteristics of the genus *Corynebacterium* collected from the web. They are gram-positive, club-shaped bacteria, non-spore form, do not carry flagella, and are non-motile.

**Figure 8.**
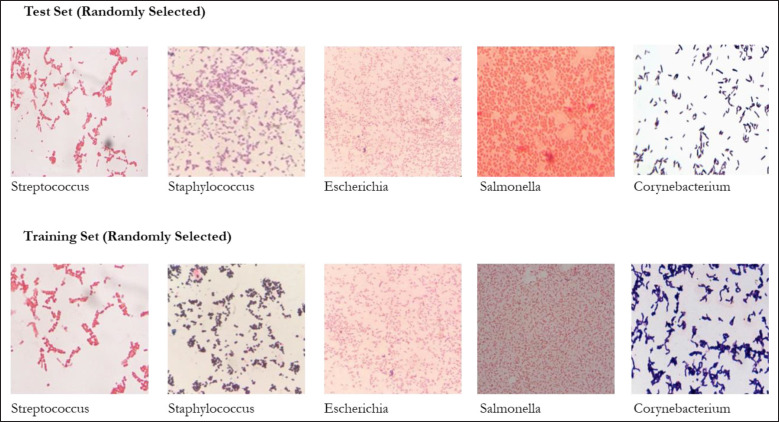
Randomly selected images (test set above five and training set below five) of the bacterial genera *Streptococcus*, *Staphylococcus*, *Escherichia*, *Salmonella*, and *Corynebacterium* fixed to run through CNN running code.

**Figure 9.**
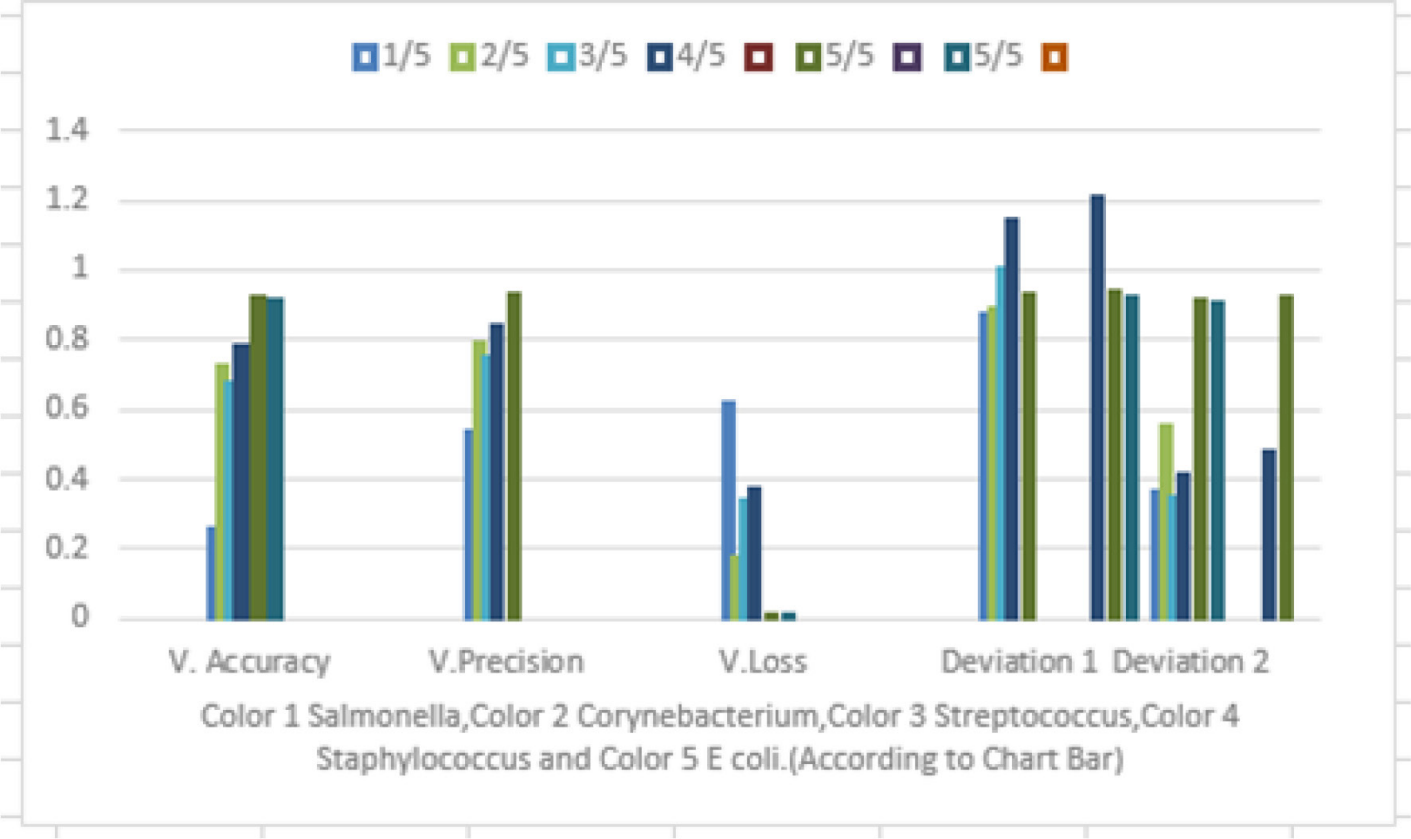
Graphical representation of the determination of the results (shown in Table 2) with five different DIBaG of bacterial genera identification using decision tree, adam optimizer, and CNN under DL technique. Legend: *V* = Value.

After collection, a preliminary dataset was prepared (Table 1) (Figs. 3–7) comprising five taxonomically and morphologically distinct bacterial genera. The images given in Figure 8 were permutated as a test and training set. As per the technique of bacterial genus identification, the model for feeding data was found to be very effective for this study (Fig. 9).

The accuracy rate of DIBaG of five different bacterial genera (*Corynebacterium, Escherichia, Salmonella, Staphylococcus and Streptococcus*) was determined as suitable and effective for the morphological shape and staining characteristics. Among the 1–5 epochs of the DL technique (Fig. 9), the accuracy rate of bacterial genera identification of *Staphylococcus* was graded 1 (92.20%), *Streptococcus* graded 2 (91.41%), *Escherichia* graded 3 (90.69%), *Corynebacterium* graded as 4 (83.55%), and *Salmonella* was graded as 5 (77.40%). To more accurately support this result, neural network performance for identification of DIBaG microscopy datasets, testing on the remaining image dataset, and repeating with other train/ test combinations may well be tried. Due to the complexity of the model and more significant variability, the variability of the number of layers utilized in this study may show little to no gain in accuracy. It should be emphasized that there are several ways to alter network complexity, such as by adding or removing layers, all of which may be worthwhile investigating. For a specific test dataset, CNN’s training variance is slight, still not zero, demonstrating that the network training approach finds similar but distinct minima with different (random) initializations on the same training data.

There may be numerous species or cell kinds, each with some physical similarities. Therefore, it is essential to consider whether these parallels can be used to reduce the requirements for neural network training. Because of how well they work, as shown here, and how easy they are to use, CNNs are likely to be used more often in biological image analysis.

The accuracy rate of five different bacterial genera DIBaG *Corynebacterium, Escherichia, Salmonella, Staphylococcus* and* Streptococcus* isolates revealed a smaller scale variation as per validation through various ML models and feature extraction, and this might be due to the variation of the test dataset and training dataset, computer converter type, morphology, the concentration of bacterial genera and staining characteristics, and also the quality of the bacterial genera images. The results of this research are partly supported by the authors of several reports [1,3,11,22,23]. Bacterial species are classified based on bacterial colony morphology instead of bacterial size and shape using the FV-SIFT, FV-CNN, and FC-CNN of the DL method. However, this study covered bacterial genera identification based on morphological features using the Grams staining technique, followed by five different DIBaG of *Corynebacterium, Escherichia, Salmonella, Staphylococcus* and* Streptococcus* with the DL technique of CNN, decision tree, Adam Optimizer, and feature extraction, through which the bacteriologists could be able to use the identification better than the previously identified traditional [23] techniques. The other previous investigators also used the DL techniques with the other bacteria [23] performed with Myxobacteria and the SVM technique with CNN and identified the bacteria with an average accuracy rate of 77.24, while this research identified the bacterial genera with the highest accuracy rate of 92.20% for the bacterial genus *Staphylococcus* (Table 2 and Fig. 9). A model using stereomicroscopically captured images of the fruiting bodies at the genus level was introduced by the automated identification of Myxobacterial genera using CNN [23]. This model eliminates the need to prepare microscopic slides of vegetative cells or myxospores and can be achieved without them. The research team [11] carried out research on a deep framework for bacterial image segmentation and classification to automatically identify bacteria, classify regions of bacterial colony images, and correspond to them across different images from different contexts. The bacterial images were segmented into regions covering the bacterial colonies, agar, plate, and various border artifacts through unsupervised DL and the convolutional deep belief network technique for a deep representation of small image patches. The study results with five different DIBaG *Corynebacterium, Escherichia, Salmonella, Staphylococcus* and* Streptococcus* were less supported by the above-mentioned investigators. However, this study needs further clarification after inclusion and focusing future research on this work. This is the first preliminary report in the context of Bangladesh.

The performance of the neural network for the identification of five bacterial genera using DIBaG microscopy datasets may be checked on the remaining image dataset and repeated with various train/test combinations to verify these results. The variability in the number of layers used in this study may show little to no gain in accuracy due to the complexity of the model and greater variability. A network’s complexity can be altered in a number of ways, including by adding or removing layers. It may be worthwhile to investigate each of these options. The network training method finds patterns that are similar to each other but still different. This is shown by the fact that CNN’s training variance for the given test dataset is small but not zero.

As per Figure 9, the depicted value determined as *Salmonella*, *Corynebacterium*, *Streptococcus*, *Staphylo-coccus*, and *Escherichia* instead of *Streptococcus*, *Staphylococcus*, *Escherichia*, *Salmonella*, and *Coryne-bacterium* used as five bacterial genera DIBaG where the graphical representation shows that in value accuracy 1 movement directed their linear regression value corresponding to *X* and *Y* axis. Similarly, value precision which is known as value accuracy 2 shows the remaining bacterial genera movement according to previous Table 2. There is a value loss bar in this chart to show the loss of accuracy per epoch according to accuracy 1 and accuracy 2. Now deviations 1 and 2 show the shifting value of 5 bacterial genera which are moving slowly in the medium. At that training, dataset-shifting is calculated in this graph (Fig. 9). DIBaG of bacterial genera movement also shows recall to record this case correctly. Excel is being used for this case to explain the whole procedure according to the DIBaG image surroundings contamination case and linear regression, shifting is shown in this graph respectively.

**Table 2.** Determination of results with five different DIBaG of bacterial genera identification using decision tree, adam optimizer, CNN, and confusion matrix under DL technique.

EPOCH	Value accuracy 1	Value precision (value accuracy 2)	Value loss	Deviation
1/5	0.2513	0.5348	0.6129	0.2513 ± 0.6129
2/5	0.7167	0.7867	0.1709	0.7167 ± 0.1709
3/5	0.6703	0.7431	0.3314	0.6703 ± 0.3314
4/5	0.7740 (*Salmonella*)	0.8355 (*Corynebacteria*)	0.3650	0.7740 ± 0.3650 (*Salmonella*) 0.8355 ± 0.3650 (*Corynebacteria*)
5/5	0.9141 (*Streptococcus*)	0.9220 (*Staphylococcus*)	0.0082	0.9141 ± 0.0082 (*Streptococcus*) 0.9220 ± 0.0082 (*Staphylococcus*)
5/5	0.9069 (*Escherichia*)	0	0.0082	0.9069 ± 0.0082 (*Escherichia*)

The limitations of the method described here served as a catalyst for additional investigation into computer science-based methods supporting bacteriological diagnostics of genera *Corynebacterium, Escherichia, Salmonella, Staphylococcus* and* Streptococcus*, as well as any other bacterial genera or species. For specific genera and species of bacteria that could not be accurately identified up until now, the approach may be verified by increasing the correctness and sensitivity of the classifiers already in use or by developing and implementing new ones. The results of experiments on the classification of images of specific bacterial species using a computer-based DL classifier demonstrate the efficacy of the proposed technique. It could be possible to enlarge the number of studied images and get even better identification results. Therefore, it could be necessary to enhance the procedure and carry out additional related studies.

## Conclusion

The study concluded that the state-of-the-art texture recognition method for the problem of classifying bacterial genera as DIBaG, e.g., *Corynebacterium, Escherichia, Salmonella, Staphylococcus* and* Streptococcus,* was successfully performed with the highest validity and accuracy rate. However, the accuracy rate of the five different genera DIBaG revealed a smaller scale variation as per validation through the feature extraction, and this might be due to the variation of the test dataset and training dataset, morphology, concentration, and staining characteristics, and also the quality of the bacterial genera images. A new dataset of picture data was provided by the study to identify a bacterial genera classifier, allowing for an evaluation of this approach and comparison with other approaches. According to the results of the studies, bacteriologists may successfully apply the best strategy in their day-to-day work for the identification and diagnosis of the proper treatment. The literature study is, to date, the first report in both the Bangladeshi and international contexts. It, therefore, calls for a thorough investigation encompassing additional bacterial genera and other criteria for classification. The focus of future studies will be on expanding the database and incorporating information about color dispersion into the researched method. This should increase the precision with which these sorts of DIBaG and similar are recognized.

## List of Abbreviations

CNN, Convolutional neural networks; DIBaG, Digital image bacterial genera; DL, Deep learning; ML, Machine learning; PCR, Polymerase chain reaction; SVM, Support Vector Machine.
